# Antioxidant, antidiabetic effects and polyphenolic contents of propolis from Siirt, Turkey

**DOI:** 10.1002/fsn3.3958

**Published:** 2024-01-09

**Authors:** Eda Ören, Salih Tuncay, Yunus Emre Toprak, Muhammet Fırat, İsra Toptancı, Ömer Faruk Karasakal, Mesut Işık, Mesut Karahan

**Affiliations:** ^1^ Department of Food Technology, Vocational School of Health Services Uskudar University Istanbul Turkey; ^2^ Department of Biotechnology Graduate Institute, Bilecik Şeyh Edebali University Bilecik Turkey; ^3^ Istanbul Food Control Laboratory Istanbul Turkey; ^4^ Department of Medical Laboratory, Vocational School of Health Services Uskudar University Turkey; ^5^ Department of Bioengineering, Faculty of Engineering Bilecik Şeyh Edebali University Bilecik Turkey

**Keywords:** antioxidant activity, Propolis, radical scavenging, α‐amylase, α‐glycosidase

## Abstract

Propolis, a natural product collected by honeybees from various plant sources, has gained significant attention due to its diverse bioactive compounds and potential therapeutic properties. To further explore its contents and biological activities, this study aimed to analyze the phenolic compounds in Siirt propolis extracts obtained using different solvents, namely ethanol, water, and ethanol‐water mixtures. The primary objective of this research was to investigate the phenolic profile, as well as the antidiabetic and antioxidant activities of the propolis extracts. Chemical profiling of extracts was performed using LC–MS/MS. The antioxidant potential of the propolis extracts was evaluated through free radical scavenging methods, including DPPH and ABTS assays. As a result of these analyses, propolis extracts showed moderate radical scavenging potential with 13.86%–35.72% for DPPH and 33.62%–62.50% for ABTS at a concentration of 30 μg mL^−1^, respectively. This radical scavenging potential of the extracts sheds light on its ability to combat oxidative stress, which is implicated in the development of diabetes, and its potential effects on cellular health. Additionally, the study assessed the antidiabetic properties of the propolis extracts by examining their inhibition effects on α‐amylase and α‐glycosidase enzymes. Extracts with high phenolic content showed a high inhibitory effect against α‐glucosidase with an IC_50_ of 5.72 ± 0.83 μg mL^−1^. This research provided significant findings regarding the potential use of propolis in the treatment of diabetes and related metabolic disorders.

## Graphical abstract

The primary objective of this research was to investigate the phenolic profile, as well as the antioxidant and antidiabetic activities of the propolis extracts. Chemical profiling of extracts was performed using LC–MS/MS. A correlation heatmap based on assessing the relationship between variables.

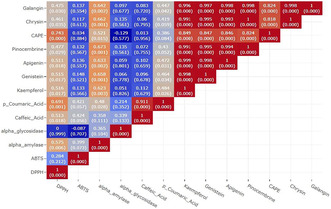

## 1 INTRODUCTION

Propolis is produced as a result of honey bees mixing the resin and various substances they collect from the trunk, leaves, shoots, and buds of trees such as oak, pine, fir, chestnut, elm, and willow with their secretions and stored by accumulating (Ghisalberti, 1979). Propolis, in colors ranging from dark yellow, green, red, and brown, depends on the secondary metabolite such as flavonoids and phenolics, the area in which it is collected and its age. It has a unique smell thanks to the essential oils it contains. The existence of propolis and its use among humans date back to ancient times (Çelemli et al., 2011). Recently, it has been widely researched in the world in terms of its chemical composition and biological properties (Stojanović et al., 2020).

Containing biologically active components, propolis shows antifungal, antibacterial, antiparasitic, antiviral, antidiabetic, hepatoprotective, antioxidant, and immunomodulatory activities (de Freitas et al., 2017; Kocot et al., 2018; Santos et al., 2020). A study on propolis reported that its antioxidant activity decreased the likelihood of cataracts in rat pups (Pessolato et al., 2011). Furthermore, a separate study has reported that propolis expedites tissue regeneration by stimulating the synthesis and release of glycosaminoglycans. Additionally, various other biological properties, including hepatoprotective, antitumoral, and cytostatic activities, have been elucidated (Oryan et al., 2018; Wagh, 2013; Xuan et al., 2014). In addition, the total phenolic and flavonoid components in propolis extracts, their phenolic profile, and biological activities may vary according to many factors such as geographical origin of the sample, harvest season and method, bee species, and solvent used for extraction (Marcucci, 1995; Touzani et al., 2021; Woźniak et al., 2023).

Flavonoids, phenolic substances, and aromatic acids are among the important chemical classes of propolis and its chemical composition includes important compounds such as pinocembrin, chrysin, rutin, apigenin, catechin, naringenin, quercetin, galangin, luteolin, kaempferol, and myricetin. It also has amino acids, inorganic compounds, and enzymes (acid phosphatase, glucose‐6‐phosphatase, succinic dehydrogenase, and adenosine triphosphatase). At the same time, propolis contains small molecules such as benzoic acid, myristic acid, benzyl alcohol, caffeic acid, vanillin, cinnamic acid, acacetin, kaempferide, and isovanillin. They also contain minerals (I, K, Mg, Cu, Zn, Ca, Na, Mn, and Fe), vitamins (B1, B2, B6, C, and E), and fatty acids, which are very important and necessary for human health (Marcucci, 1995; Woźniak et al., 2023). Due to its intense chemical content, it is important in terms of its potential to be used and developed in health, pharmaceutical production, the cosmetics industry, the food industry, and even in different fields (Devequi‐Nunes et al., 2018; Doğan & Hayoğlu, 2012).

The antioxidant activities and components of propolis extracts were studied with various extraction solvents. The results of these studies showed that ethanol was more effective than water for the extraction of phenolic contents and that ethanol extracts had greater antioxidant activity than water. However, there are few studies evaluating the phenolic profiles of different ethanol/water solvents and propolis extracts (Sun et al., 2015).

The goal of this study is to compare the phenolic content profiles of Siirt Propolis (SP) extracts obtained using water and ethanol by LC–MS/MS and characterize them, and to determine their antioxidant and antidiabetic effects by various biological activity measurements.

## 2 MATERIALS AND METHODS

### 2.1 Chemicals

In LC–MS/MS analysis, the following compounds were used as standards: gallic acid (97.5%), quercetin (95%), catechin hydrate (99%), oleuropein (98%), p‐coumaric acid (98%), chlorogenic acid (96%), pinocembrin (95%), hesperetin (95%), caffeic acid (98%), myricetin (96%), kaempferol (97%), apigenin (95%), naringenin (98%), CAPE (97%), chrysin (98%), galangin (95%), genistein (98%), rutin hydrate (≥94%), formic acid, which were purchased from Sigma Aldrich. Ethanol and HPLC‐grade methanol were purchased from Merck (Germany). Stock solutions (1000 mg/L) were prepared from each phenolic standard in MeOH. Working solutions were prepared at 1 mg/L by taking appropriate portions from the stock solutions. The compounds used for antioxidant activity such as 1,1‐diphenyl‐2‐picryl‐hydrazyl (DPPH), butylated hydroxyanisole (BHA), butylated hydroxytoluene (BHT), 2,2′‐azino‐bis(3‐ethylbenzthiazoline‐6‐sulfonic acid) (ABTS), and α‐tocopherol were obtained from Sigma (Sigma–Aldrich, Germany). Dilutions were carried out employing automated pipettes and precision glass volumetric flasks. All remaining chemicals utilized were of analytical grade and sourced from Sigma–Aldrich or Merck. The same propolis sample was collected as 150 g from areas of Siirt/Türkiye. It was collected in the summer of 2021 and stored at −18°C until further analysis.

### 2.2 Preparation of propolis solvent extracts

Propolis sample was gathered from the Siirt province in the Southeastern Anatolia region of Turkey, followed by their preservation and desiccation in a light‐protected environment before further processing. Extractions were carried at different solvent systems such as ethanol, water, and ethanol‐water (80:20, 60:40, 40:60, 20:80, *v/v*).

For ethanol extraction, 10 g of liquid nitrogen‐dried propolis was ground into a fine powder in a blender and mixed with 100 mL ethanol via a magnetic stirrer for 24 h. After 24 h, it was refluxed more than 1 h at 65°C. Then the ethanol extract was filtered over the Whatman No. 1 paper. The ethanol solvent was removed via a rotary evaporator (Heidolph Hei‐Vap Adv ML) at 40°C to obtain a dry extract. The dried ethanolic extract was stored in a plastic bottle at −20°C until further use.

For water extraction, 10 g of liquid nitrogen‐dried propolis was ground into a fine powder using a blender and then mixed with 100 mL of water and stirred with a magnetic stirrer for 24 h to extract water. After 24 h, it was refluxed 1 h at 65°C. After filtering the water extract through Whatman No. 1 paper, the resulting filtrates were frozen at −84°C in an ultra‐low temperature freezer (Sanyo, Japan). They were then lyophilized in a lyophilizer under 5 mm Hg pressure at −50°C. The dried water extract was then stored in a bottle at −20°C until it was ready to be used.

For ethanol‐water extractions, 10 g of liquid nitrogen‐dried propolis sample was ground into a fine powder in a blender and mixed with 100 mL ethanol‐water (80:20, 60:40, 40:60, 20:80, *v/v*) by magnetic stirrer for 24 h. After 24 h, it was refluxed at 65°C for 1 h. Then the extracts were filtered with Whatman No. 1 paper. The ethanol solvent was first removed from the rotary evaporator after each extraction. Then, the remaining water phase was lyophilized (such as water extraction). The dried ethanol‐water extracts were placed separately in a bottle and stored at – 20°C until used.

### 2.3 Instruments and chromatographic conditions

A liquid chromatograph‐mass spectrometer (LC–MS/MS) was used to analyze 18 distinct phenolic compounds. A Shimadzu Nexera X2 UHPLC (Shimadzu, Japan) liquid chromatograph equipped with an InertSustain Swift C18 (2.1 mm*100 mm, 3 μm) column was used, and the system was coupled to an 8050 triple quadruple detector (Shimadzu, Japan) controlled by LabSolution 5.60 SP2 software. Ultra‐pure water was prepared on a Milli‐Q IQ 7000 system (Millipore Company, USA). Propolis samples (SP1–SP6) were extracted by the Reflux Mechanism.

### 2.4 Determination of LC–MS/MS analysis conditions

Binary gradient LC‐40D XS‐ Autosampler Model SIL‐40C XS‐ Oven Model CTO‐40S‐Shimadzu 8050 model liquid chromatography device LC–MS/MS analysis of the samples was performed by triple quadrupole MS/MS detector. The elution gradient was established with mobile phase A (water and 0.1% formic acid) and mobile phase B (methanol and 0.1% formic acid). The gradient elution program started with 20% B for 0 min, increasing linearly from 50% for 8 min, then to 95% B at 12 min. Finally, the system returned to its original conditions over 0.1 min and equilibrated for 3 min (total run time 15 min). Multiple reaction monitoring processing (MRM) mode was used to quantify the analysis. Two applications were made for each compound analysis in the experiment. The first was performed for quantitative results and the second was analyzed for confirmation. The optimized MS parameters for phenolic compounds are given in Table 1. Optimum Electrospray Ionization (ESI) parameters; 350°C interface temperature, 250°C DL temperature, 400°C nebulizer gas (nitrogen) temperature, 3 L/min, gas flow 15 L/min, drying gas flow was determined.

**TABLE 1:** MS parameters for the tested compounds.

Compound	Precursor	Product	CE	Polarity
Gallic acid	168.9	125	15	Negative
Catechin hydrate	290.8	139	−13	Positive
	290.8	123	−21	Positive
Chlorogenic acid	353.3	191.3	17	Negative
	353.3	111	32	Negative
Caffeic acid	178.85	135.05	16	Negative
	178.85	89.2	33	Negative
p‐Coumaric acid	162.95	93	28	Negative
	162.95	119.5	15	Negative
Hesperidin	609	301.2	25	Negative
Rutin	608.9	300	36	Negative
	608.9	271.05	55	Negative
Oleropin	539.3	275.1	21	Negative
	539.3	306.9	21	Negative
Myricetin	317	151.2	24	Negative
	317	179.2	12	Negative
Naringenin	272.8	153	−24	Positive
	272.8	147.1	−21	Positive
Quercetin	300.8	179	18	Negative
	300.8	151	21	Negative
Kaempferol	285.2	116.9	45	Negative
	285.2	93	36	Negative
	285.2	182	49	Negative
	285.2	227.2	33	Negative
Apigenin	269.1	117	33	Negative
	269.1	149.2	22	Negative
Genistein	269.1	117.1	48	Negative
	269.1	106.9	30	Negative
Pinocembrin	254.9	213.1	19	Negative
	254.9	151.2	21	Negative
CAPE	282.85	135.05	25	Negative
	282.85	161	23	Negative
	282.85	179.05	18	Negative
Chrysin	252.8	209.15	22	Negative
	252.8	143.1	26	Negative
	252.8	62.95	31	Negative
Galangin	269.2	213.1	24	Negative
	269.2	227	25	Negative

### 2.5 Sample preparation

For phenolic component analysis, Touzani et al. (2021) was followed. 0.01 g of propolis sample (with an accuracy of 0.001 g) was weighed into a 50 mL centrifuge tube, 15 mL of methanol was added to it. It was kept in ultrasonic for 20 min. It was then centrifuged at 4000 rpm for 10 min. After centrifugation, make up to 50 mL of methanol. It was passed through filter paper and passed through a 0.45 μm filter tip, taken into a vial to give LC–MS/MS, and the measurement was made. The experiments were conducted in triplicate (*n* = 3), and the outcomes were presented as the mean value accompanied by the standard deviation.

### 2.6 Method validation parameters

The validation parameters were determined to be linearity, precision (reproducibility and, recovery), the limits of detection (LOD), and quantification (LOQ) experiments. The signal‐to‐noise ratio was determined by measuring the signal‐to‐noise ratio by injecting until the S/N ratio was 3 for LOD and 10 for LOQ. The linearity for the phenolic compounds at the concentration levels of 0.1, 0.2, 0.5, 0.75, 1, and 2 mg/kg was evaluated by injection of six calibration standards in triplicate. The correlation coefficient obtained was more remarkable than 0.995 for all analyses (*r* > .995). Method accuracy and precision were evaluated using spiked blanks. These were obtained by recovery studies performed on samples spiked at 0.1, 0.2, and 0.75 mg/kg. The recoveries and RSDs at three levels and LOQs for each phenolic compound studied are shown in Table 2. Overall, the average recoveries for the LC–MS/MS method were in the range of 80%–102%. The relative standard deviations (RSDs) ranged from 9% to 12%. Based on the results, it is evident that the suggested techniques demonstrate accuracy and precision.

**TABLE 2:** Antioxidant phenolic content's uncertainty and validation parameters.

Compounds	*R* ^2^	LOD (μg/kg)	LOQ (μg/kg)	Reproducibility RSD (%)	Repeatability RSD (%)	Recovery (%)
				(100‐200‐750 μg/kg)	(100‐200‐750 μg/kg)	(100‐200‐750 μg/kg)
Gallic acid	.999	0.063	0.09	13‐12‐11	15‐14‐12	88‐89‐97
Catechin hydrate	.999	0.028	0.1	14‐11‐11	15‐13‐13	80‐88‐93
Chlorogenic acid	.999	0.04	0.1	13‐13‐12	14‐14‐12	86‐89‐91
Caffeic acid	.999	0.036	0.08	13‐12‐11	13‐12‐11	90‐92‐97
p‐Coumaric acid	.999	0.042	0.07	13‐11‐11	19‐17‐16	87‐91‐91
Hesperidin	.999	0.03	0.09	14‐13‐12	11‐11‐11	91‐93‐101
Rutin	.999	0.06	0.1	13‐11‐11	14‐12‐11	86‐87‐90
Oleropin	.999	0.02	0.08	12‐12‐11	14‐12‐11	86‐90‐96
Myricetin	.999	0.041	0.1	13‐12‐11	14‐11‐11	85‐96‐94
Naringenin	.999	0.06	0.09	14‐13‐12	15‐12‐11	80‐87‐99
Quercetin	.999	0.04	0.1	14‐13‐11	15‐13‐11	85‐93‐99
Kaempferol	.999	0.02	0.08	14‐13‐12	14‐12‐11	85‐90‐95
Apigenin	.999	0.06	0.1	13‐12‐11	14‐12‐11	87‐90‐98
Genistein	.999	0.035	0.08	11‐11‐11	11‐11‐11	93‐93‐97
Pinocembrin	.999	0.032	0.09	13‐12‐11	15‐14‐12	88‐89‐97
CAPE	.999	0.038	0.09	12‐11‐10	14‐13‐12	99‐100‐102
Chrysin	.999	0.037	0.09	13‐12‐11	15‐12‐10	87‐89‐97
Galangin	.999	0.02	0.01	15‐14‐12	16‐13‐11	86‐88‐93

### 2.7 Determination of antioxidant and antidiabetic activities of Propolis extracts

#### 2.7.1 DPPH radical removal potential

The DPPH free radical scavenging potential was assessed using the Blois method (Blois, 1958; Kavaz Yüksel et al., 2021). A freshly prepared solution of DPPH (10^−3^ M) was utilized as the free radical source. The samples were pipetted into test tubes at concentrations of 10, 20, and 30 μg/μL in a total volume of 1 mL, followed by the addition of 96% ethanol to complete the final volume to 1.5 mL. Subsequently, 0.5 mL of the freshly prepared DPPH solution was added and mixed using a vortex. After incubating for 30 min, the percentage of radical scavenging activity was expressed by measuring the absorbance at 517 nm.

#### 2.7.2 ABTS radical removal potential

The ABTS radical scavenging potential was evaluated following the method introduced by Re et al. (Re et al., 1999). ABTS radicals were generated by combining persulfate (2.45 nM) solution with a prepared ABTS (7 mM) solution. Controls were set up using 1000 μL of ABTS radical solution and 500 μL of 96% ethanol. Next, different concentrations (ranging from 10 to 40 μg mL^−1^) of the samples were mixed with 1 mL of ABTS radical solution, and the total volume was adjusted to 1.5 mL with 96% ethanol. After 30 min of incubation in the dark, the absorbance was measured at 734 nm with respect to the blank. The ABTS radical scavenging potential was measured and expressed as a percentage in the results.

#### 2.7.3 α‐Amylase inhibitory activity

A slightly modified α‐amylase inhibition test method, as established by Yu et al. (2012), was employed in this study. Firstly, 10 μL of α‐amylase solution (1 unit/mL in distilled water) was combined with 10 μL of samples with varying concentrations and incubated at 37.0°C for 15 min. The reaction was started by adding 500 μL of substrate, which was made from a 1% starch solution in 20 mmol/L sodium phosphate buffer (pH 6.9). The sample was then incubated for 10 min at 37°C. 300 μL of DNS reagent consisting of 12% Na‐K tartrate in 0.4 mol/L NaOH and 1% 3,5 dinitrosalicylic acid was added to stop the reaction. Subsequently, the test tubes were subjected to a boiling water bath for 10 min, followed by cooling at room temperature, and 5 mL of distilled water was added. Finally, the results of the enzymatic reaction were measured at 540 nm.

#### 2.7.4 α‐Glycosidase enzyme inhibition measurement

Analysis was performed using a modified developed method for α‐glucosidase activity determination (Xu et al., 2014). 4‐nitrophenyl β‐D‐glucopyranoside (pNPG) substrate (5 mM) was dissolved in 25 mL of phosphate buffer (0.1 M). The reaction was prepared using specific ratios of substrate (pNPG), Phosphate Buffer (5.0 mM, pH 6.9), enzyme solution in Phosphate buffer (0.15 U/mL, pH 7.4). Then, the absorbance value at 540 nm was read every minute, and at the end of 5 min, the difference between the value in the first minute and the value read in the fifth minute was taken. This experiment was repeated for 5 different inhibitor concentrations. The IC_50_ value of all substances was calculated.

### 2.8 Statistical studies

This study employed statistical analysis using the R programming language to assess the relationship between variables. Pearson correlation analysis was utilized to evaluate the association between two variables. The results were exhibited as mean ± standard error of the mean (95% confidence intervals). Differences between data sets were considered statistically significant when the *p*‐value was less than .05.

## 3 RESULTS

### 3.1 Phenolic contents of propolis

The assessment of total phenolic and flavonoid contents was conducted for Siirt Propolis. Investigating the solubility and biological effects of propolis, specifically its antioxidant and antidiabetic properties, involved characterizing the phenolic compounds present in Siirt propolis extracts. These extracts were obtained using different solvents, including ethanol, water, and ethanol/water mixtures with varying ratios (20/80, 40/60, 60/40, and 80/20, v/v). The characterization was performed comparatively using LC–MS/MS. For this, 18 standard substances: “catechin, naringenin, gallic acid, caffeic acid, chlorogenic acid, p‐coumaric acid, hesperidin, rutin, oleuropein, myricetin, quercetin, kaempferol, genistein, apigenin, pinosembrin (racemic), CAPE, chrysin, and galangin” were selected to detect phenolic compounds in the extracts.

In addition, the extraction process was refluxed by increasing the temperature and time controlled. The highest extraction efficiency was obtained in ethanol, and it was observed that the yield reduced as the amount of water in the solvent was increased. Extraction yields are shown in Table 3. Especially in ethanol, phenolic compounds “caffeic acid, kaempferol, genistein, apigenin, pinosembrin (racemic), CAPE, chrysin, galangin” were found to be over 100 ppm. It was observed that phenolic compound ratios decreased as the amount of water increased, except for caffeic acid. Major phenolic compounds detected in SP1 extract: caffeic acid, kaempferol, genistein, apigenin, pinocembrin (racemic), CAPE, chrysin, galangin; Major phenolic compounds detected in SP1.1 extract: pinocembrin (racemic), chrysin, galangin; Major phenolic compounds detected in SP2 extract: caffeic acid, genistein, apigenin, pinocembrin (racemic), CAPE, chrysin, galangin; Major phenolic compound detected in SP3 and SP6 extracts: Caffeic acid; Major phenolic compounds detected in SP4 extract: Caffeic acid, Pinocembrin (racemic), Chrysin; Major phenolic compounds detected in SP5 extract: Caffeic acid, Pinocembrin (racemic), CAPE (Table 4).

**TABLE 3:** Propolis extraction amount and yield.

Groups	Extraction solvents	Extract amount^a^/10 g	Yield
SP1	Absolute ethanol	2.120 g	21.2%
SP1.1	The part precipitated in absolute ethanol (at room temperature)	2950 g	29.5%
SP2	80% ethanol‐water (80:20 v/v)	1.648 g	16.48%
SP3	60% ethanol‐water (60:40 v/v)	1.472 g	14.72%
SP4	40% ethanol‐water (40:60 v/v)	0.847 g	8.47%
SP5	20% ethanol‐water (20:80 v/v)	0.766 g	7.66%
SP6	100% water	0.706 g	7%

^a^ All results were calculated based on 10 g of propolis.

**TABLE 4:** Phenolic components (PC) detected by LC–MS/MS in propolis extracts.

MPC	SP1	SP1.1	SP2	SP3	SP4	SP5	SP6
Caffeic acid	711.19 ± 9.00	97.51 ± 1.26	441.76 ± 10.464	273.32 ± 12.374	124.91 ± 7.55	388.32 ± 11.54	1565.89 ± 18.476
p‐Coumaric acid	74.46 ± 3.58	9.51 ± 0.52	33.03 ± 1.38	20.41 ± 1.05	9.52 ± 0.74	32.26 ± 1.25	83.63 ± 1.80
Kaempferol	145.65 ± 3.13	14.31 ± 0.96	61.91 ± 1.41	3.76 ± 0.13	11.60 ± 1.82	11.99 ± 0.49	6.25 ± 0.61
Genistein	262.45 ± 9.34	31.56 ± 1.27	119.48 ± 7.55	0.87 ± 0.08	25.05 ± 1.58	22.99 ± 0.49	12.26 ± 1.03
Apigenin	290.20 ± 3.47	34.55 ± 0.59	128.38 ± 2.25	11.06 ± 0.32	27.81 ± 0.63	25.66 ± 0.48	14.86 ± 1.15
Pinocembrin	1439.67 ± 17.29	257.97 ± 8.72	769.20 ± 8.73	49.85 ± 0.90	149.03 ± 4.59	104.92 ± 6.46	75.19 ± 2.00
CAPE	602.43 ± 8.56	94.68 ± 1.46	288.42 ± 4.52	20.82 ± 0.74	53.08 ± 2.40	377.14 ± 5.11	22.99 ± 1.77
Chrysin	1134.62 ± 7.59	186.13 ± 5.51	603.68 ± 3.44	38.38 ± 3.97	115.95 ± 2.76	60.76 ± 2.55	35.30 ± 1.96
Galangin	835.65 ± 7.13	104.42 ± 6.96	388.12 ± 7.09	20.97 ± 0.14	61.93 ± 0.43	28.58 ± 0.74	17.35 ± 0.76
Catechin	ND	ND	ND	ND	ND	ND	ND
Naringenin	26.37 ± 0.75	3.26 ± 0.06	10.72 ± 0.22	1.27 ± 0.19	2.57 ± 0.08	5.16 ± 0.06	6.24 ± 0.10
Gallic acid	0.83 ± 0.05	ND	0.33 ± 0.04	0.48 ± 0.04	ND	0.47 ± 0.02	ND
Chlorogenic acid	1.23 ± 0.11	0.15 ± 0.03	1.86 ± 0.12	0.23 ± 0.03	0.30 ± 0.02	1.46 ± 0.07	1.96 ± 0.05
Hesperetin	0.40 ± 0.01	<LOQ	0.41 ± 0.01	0.30 ± 0.02	0.10 ± 0.01	0.16 ± 0.01	0.24 ± 0.03
Rutin	15.30 ± 0.82	1.40 ± 0.14	5.33 ± 0.07	6.00 ± 0.20	1.37 ± 0.05	6.66 ± 0.06	1.96 ± 0.07
Oleuropein	13.21 ± 0.08	<LOQ	8.28 ± 0.05	87.16 ± 0.14	0.71 ± 0.02	16.57 ± 0.20	ND
Myricetin	ND	ND	ND	ND	ND	ND	ND
Quercetin	40.56 ± 0.51	3.47 ± 0.10	15.44 ± 0.30	2.72 ± 0.16	3.09 ± 0.10	4.79 ± 0.18	1.64 ± 0.12

*Note*: Phenolic compounds amount were expressed as μg mL^−1^.

Abbreviation: ND, not detected.

**FIGURE 1:**
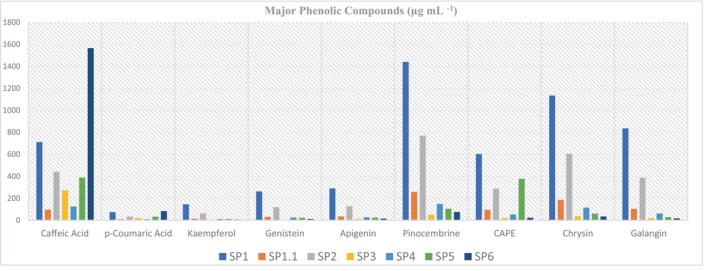
Major phenolic compounds according to LC–MS/MS results (μg mL^−1^).

Among the extracts obtained from all solvent systems, the most common phenolics were caffeic acid, pinocembrin, chrysin, CAPE, and galangin (Figure 1). Although the solubility of propolis was very low, the highest solubility and the highest content of phenolic substances were observed in absolute ethanol. In the study was obtained highest yield in ethanol extract (2.12 g/100 mL at 25°C).

### 3.2 Antioxidant and antidiabetic effect of propolis extracts

The results of the antioxidant and antidiabetic evaluations of propolis extracts are shown in Table 5. The antioxidant effect was assessed using DPPH and ABTS analyses, while the antidiabetic effect was determined based on α‐amylase and α‐glucosidase inhibition activity.

**TABLE 5:** Radical scavenging potentials of propolis extracts and inhibition effects on diabetes‐related enzymes α‐amylase and α‐glucosidase.

Samples	DPPH^a^	ABTS^a^		α‐Glycosidase (μg mL^−1^)	
			α‐Amylase^b^	IC_50_	*R* ^2^
**SP1**	35.72 ± 4.03	62.50 ± 5.96	49.45 ± 5.18	5.72 ± 0.83	.968
**SP1.1**	17.85 ± 2.63	61.39 ± 4.87	40.49 ± 3.59	10.66 ± 1.67	.992
**SP2**	25.96 ± 3.83	33.62 ± 2.83	48.76 ± 5.09	49.50 ± 3.73	.954
**SP3**	13.86 ± 1.19	37.50 ± 3.68	21.48 ± 1.83	15.75 ± 2.37	.929
**SP4**	16.09 ± 2.03	34.05 ± 3.13	36.36 ± 3.05	ND^c^	
**SP5**	35.23 ± 3.47	40.08 ± 3.64	34.71 ± 2.08	ND^c^	
**SP6**	29.72 ± 4.23	58.19 ± 6.03	43.25 ± 3.27	27.72 ± 1.86	.946
BHT^d^	46.33 ± 2.64	88.35 ± 7.23			
Trolox^d^	95.59 ± 6.53	94.06 ± 8.35			
Acarbose^e^			94.25 ± 4.67	18.67 ± 1.32	.986

^a^ Values are expressed as percent radical scavenging particles at 30 μg mL^−1^.

^b^ Values are expressed as percent inhibition at 30 μg mL^−1^.

^c^ ND effect could not be determined.

^d^ Standard antioxidants for radical forcing (BHT, butylated hydroxytoluene, Trolox).

^e^ Glycosidase and amylase enzyme inhibitor.

Standard antioxidants showed a strong DPPH and ABTS radical scavenging potential (range 46.33%–95.59%) at a concentration of 30 μg mL^−1^. The samples exhibited radical scavenging activity in the range of 13.86%–35.72% for DPPH and 33.62%–62.50% for ABTS at a concentration of 30 μg mL^−1^, respectively. At 30 μg mL^−1^, samples **SP1** and **SP5** showed the highest DPPH radical scavenging potential at about 35%, while ABTS radical scavenging potential was observed in samples **SP1** and **SP1.1** at about 62%. In all samples, the lowest radical scavenging potential was observed in sample **SP3** for DPPH and **SP2** for ABTS.

Inhibitory effects on α‐amylase and α‐glucosidase enzymes of all propolis samples were shown (Table 5). The highest inhibitory effect on the enzymes was determined for **SP1**. At 30 μg mL^−1^, sample **SP1** had an inhibitory effect on α‐amylase enzyme with 49.45 ± 5.18%, while it had an inhibitory effect on α‐glucosidase with an IC_50_ of 5.72 ± 0.83 μg mL^−1^. While examples **SP3** showed the lowest inhibitory effect on α‐amylase, the lowest effect on α‐glucosidase was observed in **SP2**. It was determined that samples **SP4** and **SP5** did not have any effect on the α‐glucosidase enzyme.

## 4 DISCUSSION

Propolis is a natural product resulting from the mixing of bee secretions with botanical exudates and Phenolic components in its content are vital components of the human diet. It is defined as an extremely complex resinous material containing biologically active molecules with antioxidant, antibacterial, antiparasitic, antiviral, hepatoprotective, antifungal, antidiabetic, and immunomodulatory activities (de Freitas et al., 2017; Kocot et al., 2018; Santos et al., 2020). Phenolic contents (flavonoids), common especially in its composition, are plant metabolites commonly distributed in the plant kingdom. Recent focus of attention on phenolic acids stems from their potential protective role against oxidative damage diseases (DM, coronary heart disease, stroke, and cancers) through the ingestion of fruits and vegetables. In addition, it was reported that the application of propolis extracts has been tried in therapies metabolic diseases, inflammation, and against cancer, since propolis is rich in flavonoids such as kaempferol, genistein, apigenin, chrysin, and cinnamic acid derivatives. Studies on animal and cellular models indicate that propolis can regulate the accumulation of AGEs, oxidative stress, and inflammation in adipose tissue, all of which contribute to insulin defects (Kitamura, 2019). Studies have reported that the primary components of propolis are phenolic compounds, which consist of caffeic, ferulic, p‐coumaric, and cinnamic acids. The pharmacological benefits of propolis are associated with its phenolic compounds and flavonoid skeleton structure (Gülçin et al., 2010; Kumazawa et al., 2004). Phenolic contents with and without flavonoid structure are widely included in the content of propolis produced by bees in different regions of the world (Jordan, Brazil, Turkey, etc.), and solutions obtained from propolis are used extensively in traditional treatment methods and in folk medicine. For centuries, propolis has been used medicinally to treat infections and promote wound healing (Alencar et al., 2007; Naik et al., 2021).

The current COVID‐19 pandemic also shows that food supplements that strengthen the immune system, such as propolis, are very important. The contents of propolis produced in different regions may also contain different components. Phenolic contents share the same general structure that carries the aromatic hydroxyl functional group and form a class of approximately 8000 molecules in nature (Karaman et al., 2010).

There are uncertainties about the content of commercial products prepared from available propolis extracts. Solubility of propolis in water due to its organic structure and in ethanol due to its resinous structure is very low. In recent years, solutions (tincture) “claimed to have 20 percent propolis content” are also widely sold as food supplements.

For this reason, it is significant to obtain the contents of propolis in different regions with new techniques and to evaluate their biological activities. In our study, phenolic compounds were determined by LC–MS/MS analysis of Siirt propolis extracts obtained by using different solvents such as (ethanol, water, and ethanol‐water mixtures). While the solubility is highest in ethanol (21%), it gradually decreases in the transition to the water phase. The extract obtained a yield of 7% in the water phase.

In the study, phenol and flavonoid contents of the extracts obtained using different solvents were determined. In 100% ethyl alcohol solvent, phenolic compounds were detected CAPE 602.43 μg mL^−1^, caffeic acid 711.19 μg mL^−1^, galangin 835.65 μg mL^−1^, chrysin 1134.62 μg mL^−1^, pinocembrin 1439.67 μg mL^−1^, apigenin 290.20 μg mL^−1^, genistein 262.45 μg mL^−1^, kaempferol 145.65 μg mL^−1^, and p‐coumaric acid 74.46 μg mL^−1^ for SP1, and CAPE 94.68 μg mL^−1^, caffeic acid 97.51 μg mL^−1^, galangin 104.42 μg mL^−1^, chrysin 186.13 μg mL^−1^, and pinocembrin 257.97 μg mL^−1^ for SP1.1. In the mixture of ethanol:water (80:20, v/v) were detected phenolic compounds such as kaempferol 61.91 μg mL^−1^, genistein 119.48 μg mL^−1^, apigenin 128.38 μg mL^−1^, CAPE 288.42 μg mL^−1^, galangin 388.12 μg mL^−1^, caffeic acid 441.76 μg mL^−1^, chrysin 603.68 μg mL^−1^, and pinocembrin 769 μg mL^−1^. In the mixture of ethanol:water (60:40. v/v) were detected compounds such as pinocembrin 49.85 μg mL^−1^, oleuropein 87 μg mL^−1^, and caffeic acid 273 μg mL^−1^. Phenolic compounds were detected caffeic acid 124.91 μg mL^−1^, pinocembrin 149.03 μg mL^−1^, CAPE 53.08 μg mL^−1^, chrysin 115.95 μg mL^−1^ and galangin 61.93 μg mL^−1^ for SP4, caffeic acid 388.32 μg mL^−1^, pinocembrin 104.92 μg mL^−1^, CAPE 377.14 μg mL^−1^, and chrysin 60.76 μg mL^−1^ for SP5, and caffeic acid 1565.89 μg mL^−1^, p‐coumaric acid 83.63 μg mL^−1^, and pinocembrin 75.19 μg mL^−1^ for SP6. Pellati et al. (2011) reported, using ethanol:water (60:40–90:10, v/v) solvents, apigenin 0.02–0.44 mg mL^−1^, galangin 0.54–7.54 mg mL^−1^, caffeic acid 0.04–1.19 mg mL^−1^, kaempferol 0.03–0.39 mg mL^−1^, chrysin 0.52–705 mg mL^−1^, quercetin 0.05–0.42 mg mL^−1^, and p‐coumaric acid 0.03–1.15 mg mL^−1^. For the same phenolic substances (SP2, ethanol:water 80:20, v/v) were obtained similar results within the limit values reported by Pellati. In another study, the amounts of phenolic compounds extracted with ethanol:water (70:30, v/v) solvent were apigenin (0.03 mg mL^−1^), gallic acid (0.04 mg mL^−1^), caffeic acid (0.59 mg mL^−1^), kaempferol (0.61 mg mL^−1^), chrysin (0.14 mg mL^−1^), naringenin (0.15 mg mL^−1^), p‐coumaric acid (2.64 mg mL^−1^), rutin (0.13 mg mL^−1^), and cinnamic acid (0.1 mg mL^−1^) were reported (Andrade et al., 2017). Phenolic compounds were isolated from environmentally friendly white bee propolis. Propolis water extracts were found to be effective on the wound healing model, and the free radical scavenging activities of the compounds isolated from these extracts (fraxetin, apigenin, galangin, etc.) were found to be high (Necip et al., 2023). In the study by Sun et al. (2015), it was found that there are characteristic components of caffeic acid, pinocembrin, chrysin, and galangin in 75% ethanol:water solvent. It has been observed that our study is similar to the previous studies (Andrade et al., 2017; Pellati et al., 2011; Sun et al., 2015).

Devequi‐Nunes et al. (2018) conducted an evaluation of propolis extracts obtained through ethanolic extraction and supercritical extraction from three different propolis species (brown red and green) collected in various regions of Brazil (specifically, the state of Bahia). The study involved the determination of phenolic compounds, flavonoids, in vitro antioxidant activity (DPPH), as well as luteolin, ferulic acid, and catechin content in the propolis extracts. The results indicated that the ethanol extracts yielded the most favorable outcomes, displaying significant selectivity for extracting antioxidant compounds (Devequi‐Nunes et al., 2018). On the other hand, Woźniak et al. (2023) investigated the chemical composition and biological properties of propolis extracts obtained from different regions in Poland. The study revealed moderate DPPH free radical scavenging activity ranging from 29.22% to 35.14% at the concentration 0.1 mg mL^−1^ (Woźniak et al., 2023). In a study, antioxidant activities as well as metabolic profile of 20 propolis samples from different regions of Greece were investigated. It was found that DPPH radical scavenging activity of methanolic extracts showed versatile bioactivity ranging from 19.87% to 85.26% inhibition at 150 μg mL^−1^, while radical scavenging activity values for ABTS were 8.90%–47.60%. It was stated that samples with high total phenolic and flavonoid content showed high activity (Stavropoulou et al., 2021). There are numerous other studies in the literature that provide supportive evidence for this claim (Al‐Khayri et al., 2022; Kavaz et al., 2022; Kavaz Yüksel et al., 2021; Tohma et al., 2015). According to the results obtained from our study, higher radical scavenging activity was found at the concentration of 30 μg mL^−1^ compared to other literature studies mentioned above. In addition, samples with high phenolic content, especially **SP1** and **SP2**, exhibited higher radical scavenging activity as stated in the literature (Stavropoulou et al., 2021).

Treating diabetes mellitus (DM) involves therapeutic approaches that focus on inhibiting enzymes responsible for breaking down polysaccharide molecules into simpler sugars or monosaccharides. This inhibition, particularly of α‐glycosidase and α‐amylase enzymes, slows down the digestion and absorption of simple carbohydrates in the intestine. Consequently, it helps regulate postprandial blood glucose levels, contributing to the treatment of DM. Certain clinically recommended medications, such as acarbose and voglibose, act as α‐glycosidase inhibitors, facilitating the control and treatment of DM (Karagecili et al., 2023). In a study, antioxidant and antidiabetic properties of Berdav propolis were determined. Propolis extract showed an inhibitory effect against α‐glycosidase with IC_50_ values of 3.7 μg mL^−1^ (Karagecili et al., 2023). In another study, they examined the inhibitory effects of natural phenolic compounds on α‐glycosidase and α‐amylase enzymes in vitro. These compounds showed significant inhibition against α‐amylase and α‐glycosidase with IC_50_ values ranging from 137.36 to 737.23 nM and 29.01 to 157.96 nM, respectively. It has also been reported that caffeic acid phenethyl ester (CAPE) is a bioactive molecule of propolis and has a high inhibitory potential on these two enzymes (Karagecili et al., 2023). When the aforementioned and other literature studies were evaluated, it was stated that the inhibition of α‐glycosidase and α‐amylase increased due to the increase in phenolic content. When our results are evaluated, it can be stated that samples with high phenolic content exhibit higher inhibitory potential. In this context, other literature studies and these results support each other (Chaudhry et al., 2017; Taslimi & Gulçin, 2017).

Caffeic acid, p‐coumaric acid, kaempferol, geistein, apigenin, pinocembrin, CAPE, chrysin, and galangin are above 50 μg mL^−1^ among the 18 different phenolic compounds. In addition, SP1 and SP2 crude extracts are the samples in which the phenolic components are obtained at the highest rate, and especially the SP1 extract is much richer in flavonoids such as caempferol, genistein, and apigenin. It can be said that SP1 has the highest antioxidant levels in DPPH (35.72 ± 4.03%) and ABTS (62.50 ± 5.96%) analyses due to increased phenolic structures in the matrix and it also has an antidiabetic effect by showing the highest inhibition on the α‐amylase enzyme (49.45 ± 5.18%) and α‐glucosidase enzyme (IC_50_ of 5.72 ± 0.83 μg mL^−1^). These effects gradually decrease as the water rate in the solvent increases, but as the water rate in the solvent increases, the amount of caffeic acid (1565.89 μg mL^−1^) increases at least twice compared to the SP1 extract (Table 4).

**FIGURE 2:**
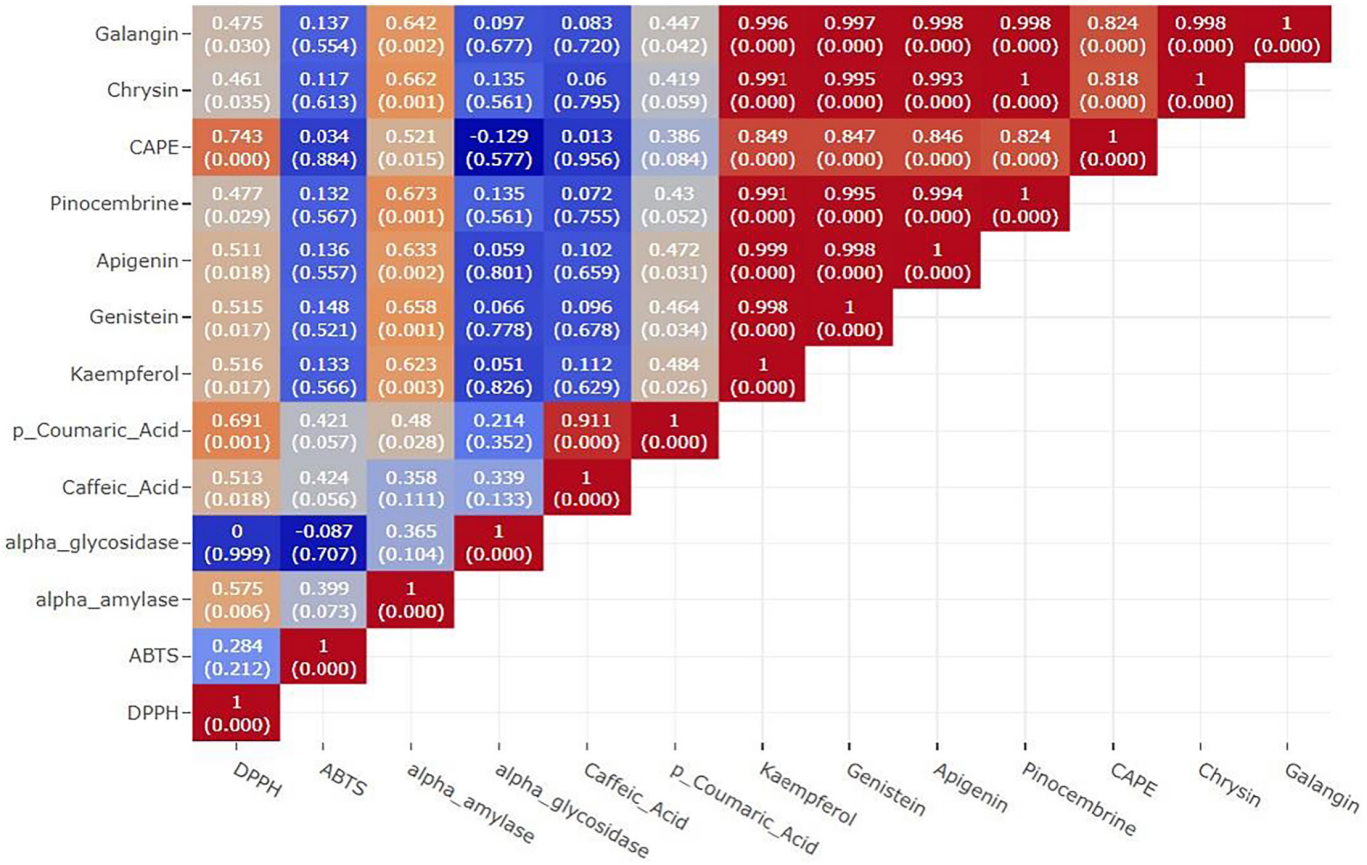
A correlation heatmap based on assessing the relationship between variables. The number in brackets in each cell represents the *p*‐values and the other represents the *R*‐value. *R*‐values are a measure of correlation strength and range from −1 to +1.

According to the results of Pearson correlation analysis (Figure 2), there is a significant positive correlation between the DPPH radical scavenging potential of all other components, especially CAPE (*p* < .001, *R* = .743) and p‐Coumaric Acid (*p* = .001, *R* = .691) in propolis. Caffeic acid and p‐Coumaric acid also showed a significant effect on ABTS radical scavenging activity. When these results are evaluated, the high radical scavenging activity in SP1, SP5, and SP6 samples can be attributed to these compounds. Genistein (*p* = .001, *R* = .658), apigenin (*p* = .002, *R* = .633), pinocembrine (*p* = .001, *R* = .673), chrysin (*p* = .001, *R* = .662), kaempferol (*p* = .003, *R* = .623), and galangin (*p* = .002, *R* = .642) compounds caused high α‐amylase inhibition effect with positive correlation results. The high α‐amylase inhibition potential in SP1, SP2, and SP6 samples can be attributed to these compounds. In addition, a positive correlation was found between the increase in DPPH radical scavenging potential of the samples and the α‐amylase inhibition effect (*p* = .006, *R* = .575). All compounds except caffeic acid and p‐Coumaric acid in the extracted content show a positive correlation with each other proportionally (*p* < .001, *R* > .800).

A study conducted in 2011 examined the anti‐radical activities of CAPE. They stated that CAPE has a good radical scavenging capacity (IC_50_: 9.8 and 3.3 mg mL^−1^, respectively) against DPPH and ABTS radicals compared to standard antioxidant compounds such as butylated hydroxyanisole (BHA), butylated hydroxytoluene (BHT), α‐tocopherol, and trolox (Göçer & Gülçin, 2011). It has also been reported that caffeic acid has the highest peroxyl radical scavenging compared to the standard antioxidant trolox. In particular, it has been shown in in vitro analyses that caffeic acid has the radical scavenging potential of ABTS (92.9% at 25 mg mL^−1^) and DPPH (92.1% at 20 mg mL^−1^) (Khan et al., 2016).

In antidiabetic studies, apigenin isolated from *Agave americana* shows an inhibitory effect (45.83% at 60 μM) against human pancreatic α‐amylase enzyme (Sahnoun et al., 2018). In another study, it was determined that kaempferol (18%) and apigenin (21%) had an inhibitory effect on the α‐amylase at 0.5 mM (Tadera et al., 2006).

As a result, when the above‐mentioned literature results and the effects of the components within the scope of this study on bioactivity were evaluated together according to Pearson correlation (Figure 2), it was seen that they were compatible with the literature studies. The results indicate that the antioxidant and antidiabetic effects of the propolis samples may vary depending on factors such as the propolis source, collection region, and extraction method. It is essential to conduct further investigations to understand the variations in the antioxidant properties of different propolis samples thoroughly.

## 5 CONCLUSIONS

Propolis, which has a natural and rich content of pollen collected by bees from the habitats around them, is of critical importance for the bees themselves, their offspring, and their hives. Due to the rich phenolics and flavonoids it contains, it is also an important food supplement for people's nutrition and strengthening the immune system. This product, which has a very rich and nutritious content, has been used as a source of healing by human beings for years. According to the results of LC–MS/MS analysis in this study, the major components determined in propolis are caffeic acid, pinosembrin, CAPE, chrysin, galangin, genistein, and apigenin. the propolis ethanol extract had the highest phenolic content and antioxidant activity and it also inhibited α‐amylase. Therefore, it can be used for antidiabetic effect. Although the phenolic content decreased due to the increase in the water phase, a significant increase in caffeic acid concentration in the propolis water extract (SP6) was observed. Compared to other phenolic compounds, the increase of caffeic acid and p‐coumaric acid in the SP6 can contribute to the DPPH radical scavenging potential. Therefore, water extracts of propolis can be used as antioxidant additives.

## AUTHOR CONTRIBUTIONS

**Eda Ören:** Methodology (equal). **Salih Tuncay:** Funding acquisition (equal); investigation (equal); methodology (equal); supervision (lead); writing – original draft (equal); writing – review and editing (equal). **Yunus Emre Toprak:** Methodology (supporting). **Muhammet Fırat:** Methodology (supporting). **İsra Toptancı:** Investigation (equal); methodology (equal); validation (lead); writing – original draft (equal); writing – review and editing (equal). **Ömer Faruk Karasakal:** Investigation (equal); methodology (supporting); writing – review and editing (equal). **Mesut Işık:** Investigation (equal); methodology (equal); writing – original draft (equal); writing – review and editing (equal). **Mesut Karahan:** Investigation (supporting); writing – review and editing (supporting).

## CONFLICT OF INTEREST STATEMENT

The authors declare no conflict of interest.

## Data Availability

The original data and supplementary materials provided in this article can be obtained from the corresponding authors.
